# Detection of DENV-2 and Insect-Specific Flaviviruses in Mosquitoes Collected From Jeddah, Saudi Arabia

**DOI:** 10.3389/fcimb.2021.626368

**Published:** 2021-02-25

**Authors:** Yuan Fang, Ernest Tambo, Jing-Bo Xue, Yi Zhang, Xiao-Nong Zhou, Emad I. M. Khater

**Affiliations:** ^1^ National Institute of Parasitic Diseases, Chinese Center for Disease Control and Prevention, Shanghai, China; ^2^ Chinese Center for Tropical Diseases Research, Shanghai, China; ^3^ WHO Collaborating Centre for Tropical Diseases, Shanghai, China; ^4^ National Center for International Research on Tropical Diseases, Ministry of Science and Technology, Shanghai, China; ^5^ Key Laboratory of Parasite and Vector Biology, Ministry of Health, Shanghai, China; ^6^ National Institute of Parasitic Diseases, Chinese Center for Disease Control and Prevention-Shenzhen Center for Disease Control and Prevention Joint Laboratory for Imported Tropical Disease Control, Shanghai, China; ^7^ Public Health Pests Laboratory, Municipality of Jeddah Governorate, Jeddah, Saudi Arabia; ^8^ Department of Entomology, Faculty of Science, Ain Shams University, Cairo, Egypt

**Keywords:** *Aedes aegypti*, Culex flavivirus, *Culex quinquefasciatus*, dengue virus, insect specific flavivirus

## Abstract

**Background:**

Mosquito-borne diseases are rapidly spreading due to increasing international travel and trade. Routine mosquito surveillance and screening for mosquito-borne pathogens can be early indicators for local disease transmission and outbreaks. However, arbovirus detection in mosquito vectors has rarely been reported in Saudi Arabia.

**Methods:**

A total of 769,541 *Aedes* and *Culex* mosquitoes were collected by Black Hole traps during routine mosquito surveillance in the first half of 2016. *Culex. quinquefasciatus* and *Ae. aegypti* were the most prevalent species observed. Twenty-five and 24 randomly selected pools of *Ae. aegypti* and *Cx. quinquefasciatus*, respectively, were screened for arboviruses by RT-PCR.

**Results:**

Dengue 2 (DENV-2) and four strains of insect-specific flaviviruses, including one of cell-fusing agent virus (CFAV) and three of Phlebotomus-associated flavivirus (PAFV) were detected in pools of *Ae. aegypti*. We also detected 10 strains of Culex flavivirus (CxFV) in pools of *Cx. quinquefasciatus*. Phylogenetic analysis using whole genome sequences placed the DENV strain into the cosmopolitan 1 sub-DENV-2 genotype, and the CxFVs into the African/Caribbean/Latin American genotype. These analyses also showed that the DENV-2 strain detected in the present study was closely related to strains detected in China in 2014 and in Japan in 2018, which suggests frequent movement of DENV-2 strains among these countries. Furthermore, the phylogenetic analysis suggested at least five introductions of DENV-2 into Saudi Arabia from 2014 through 2018, most probably from India.

**Conclusions:**

To our knowledge, this study reports the first detection of four arboviruses DENV, CFAV, PAFV, and CxFV in mosquitoes in Saudi Arabia, which shows that they are co-circulating in Jeddah. Our findings show a need for widespread mosquito-based arbovirus surveillance programs in Saudi Arabia, which will improve our understanding of the transmission dynamics of the mosquito-borne arboviruses within the country and help early predict and mitigate the risk of human infections and outbreaks.

## Introduction

Mosquitoes are the most important arthropod disease vectors in the world. They transmit malaria parasites, lymphatic filariasis nematodes, dengue virus (DENV), Chikungunya virus (CHIKV), Zika virus (ZIKV) virus, Japanese Encephalitis virus, St. Louis Encephalitis virus, Lacrosse Encephalitis virus, Eastern Equine Encephalitis virus, Western Encephalitis virus, West Nile virus (WNV), Mayaro virus, yellow fever virus, Venezuelan Encephalitis virus and many others worldwide. It is estimated that every year, approximately 700 million people are diagnosed with a mosquito-borne disease and approximately one million deaths are attributed to mosquito-borne diseases (Caraballo and King, 2014). Although the disease burden of malaria and filariasis has gradually been alleviated (WHO, 2018), the rapid spread of mosquito-borne arboviruses and associated epidemics, especially DENV, CHIKV, and ZIKV, present serious threats to public health worldwide (Roth et al., 2014). In addition, novel mosquito-transmitted arboviruses are emerging (Pybus et al., 2002).

At least four arboviruses have been detected in Saudi Arabia: DENV, Alkhurma hemorrhagic fever virus, Rift Valley fever virus, and Crimean-Congo hemorrhagic fever virus (Al-Saeed et al., 2017). DENV is currently the most prevalent. Interestingly, Saudi Arabia was traditionally a DENV-free country (Alhaeli et al., 2016); however, the recent DENV outbreaks in Jeddah, Makkah (Mecca), and Madinah in the western region (Heilman et al., 2014; Organji et al., 2017; Mohammed et al., 2018), and in Jazan in the southwestern region (Alhaeli et al., 2016; Hashem et al., 2018) suggest widespread circulation and transmission of the virus in the country. DENV-1 and DENV-2 were the most prevalent serotypes circulating during the first reported DENV outbreak in Saudi Arabia in 1994 (Fakeeh and Zaki, 2001; Zaki et al., 2008). DENV-3 was first detected in 1996 and was the dominant serotype in the dengue epidemic of 1997 (Fakeeh and Zaki, 2001). DENV-3 was not detected again for several years, but was probably circulating at low levels until it was detected again in 2004 in Jeddah (Zaki et al., 2008) and Makkah (Khan et al., 2008). Recent studies have shown that the three serotypes have been sporadically transmitted in Jeddah and Makkah (Al-Saeed et al., 2017; Organji et al., 2017). Furthermore, the DENV-4 serotype was found in serum samples from blood donors in Makkah that were collected between March 2015 and August 2016 (Ashshi, 2017), and DENV-4 infection was detected in a few cases from Jeddah in 2015 (Al-Saeed et al., 2017). Thus, all four DENV serotypes may be currently co-circulating in Saudi Arabia, and, periodical active surveillance in the *Ae. aegypti*-prone cities is needed, especially in Jeddah and Makkah, which constitute the Makkah Almokarramah (Holy) region.

The first documented DENV outbreak in Saudi Arabia was in Jeddah (289 confirmed cases) in 1994 (Fakeeh and Zaki, 2001). Jeddah is the second largest city in Saudi Arabia, and has a mean annual temperature range of 22°C–34°C, and a rainy season at the end of the year. The hot and humid climate of Jeddah is suitable for mosquito breeding. In addition, as a coastal city, Jeddah is rich in underground water and water sanitation is underdeveloped, which creates plenty of *Culex* mosquito larval habitats and is ideal for *Cx. quinquefasciatus*, the primary vector of RVFV and WNV (Franklinos et al., 2019). Owing to fresh-water scarcity, people largely depend on water storage devices as well as air-conditioning, which creates most of the *Ae. aegypti*-preferred larval water containers (natural and man-made) associated with human dwellings and activities. The city of Jeddah contains a major Saudi Arabian seaport and harbor, as well as the main airport on the Red Sea and is less than 100 km from the Holly City of Makkah. Millions of Muslim pilgrims from DENV-endemic countries travel through Jeddah to reach the Holy City of Makkah for the Holy Hajj and Umrah ceremonies every year. These pilgrims increase the chances of introducing DENV and other mosquito-borne pathogens into the city of Jeddah (Zaki et al., 2008; Alhaeli et al., 2016). The presence of high densities of competent mosquito vector in field populations, especially *Ae. aegypti*, in Jeddah puts this city at high risk of transmission of mosquito-borne viruses, especially DENV. Notably, more than 73% (3161 cases in total) of the laboratory-confirmed dengue cases in Saudi Arabia in 2015 were detected in Jeddah (Hashem et al., 2018). Moreover, the DENV antibody prevalence among the residents of Jeddah in 2015 was 47.8% (927/1939) (Jamjoom et al., 2016).

The recent studies by Alikhan et al. (2014) have observed that the relative abundance of *Ae. aegypti* in and around Jeddah is increasing (Alikhan et al., 2014), which suggests that the risk of *Ae. aegypti*-borne diseases is also increasing. Furthermore, insecticide resistance to deltamethrin was detected in field populations of *Ae. aegypti* in Jeddah and Makkah (Al Nazawi et al., 2017), making the control of this vector difficult within these cities. To the best of our knowledge, mosquito-borne disease surveillance in Saudi Arabia is currently limited to DENV, and this mainly focuses on screening serum of clinically suspected cases in endemic areas. Mosquito-based arboviral surveillance programs have not been implemented and infection rates in mosquito vectors are not routinely investigated in Saudi Arabia. It is currently unknown whether mosquito-borne arboviruses than DENV exist in Saudi Arabia. Thus, establishing mosquito-based arboviral surveillance is urgently needed to understand the dynamics of mosquito-borne disease transmission in this region. In the present study, we used mosquito samples from routine surveillance in Jeddah in the first half of 2016 to estimate the infection rates of alphaviruses, flaviviruses, and orthobunyaviruses in mosquitoes, and used molecular phylogeny to study the genotypes and the possible geographical origins of these arboviruses.

## Materials and Methods

### Mosquito Sampling

**Figure 1:**
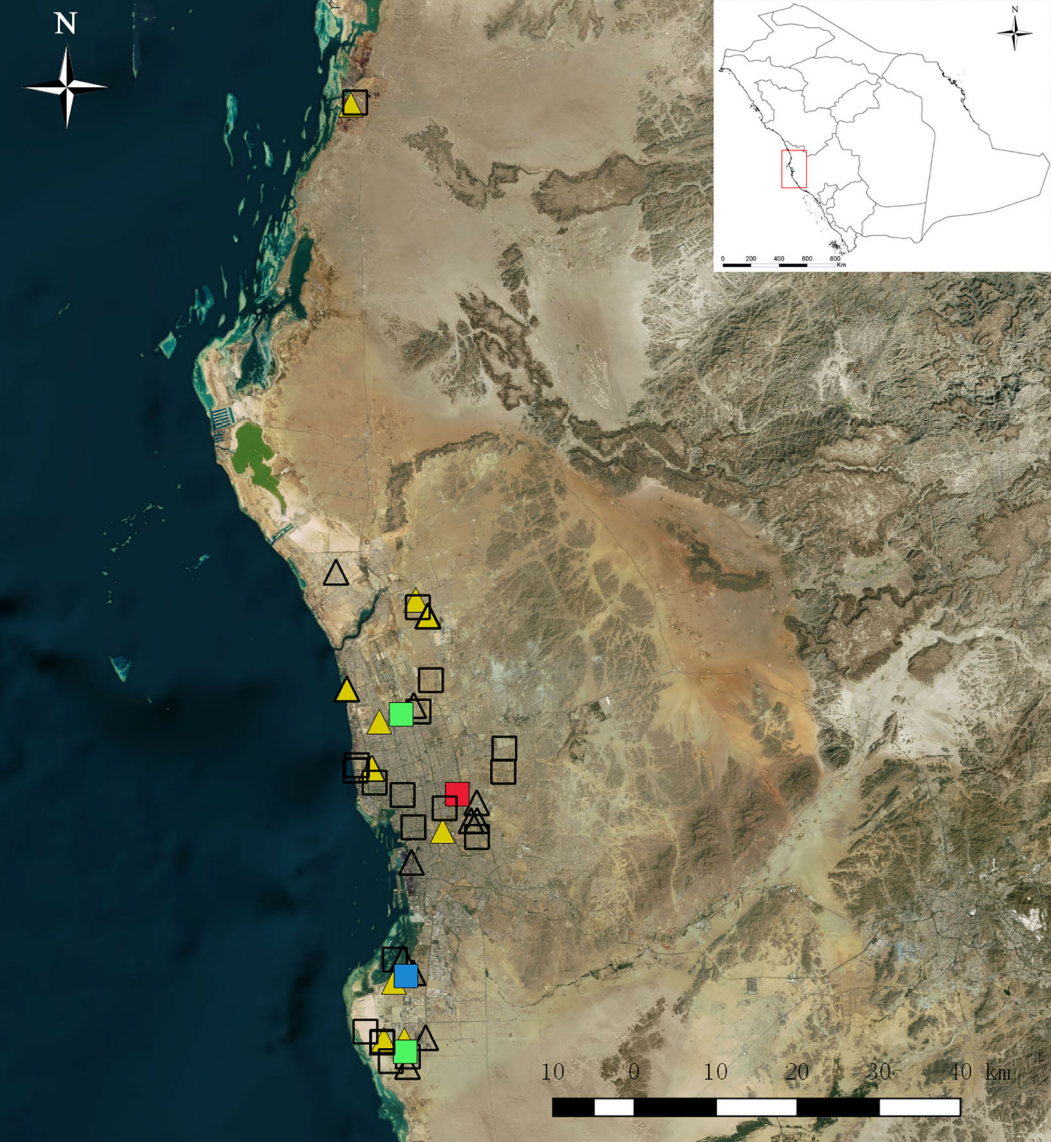
Map of mosquito collection sites in 2016 in Jeddah, Saudi Arabia. Squares represent collection sites of *Aedes aegypti*, triangles represent sites of *Culex quinquefasciatus*. Color symbols filled with red, yellow, green, and blue represent dengue virus, Culex flavivirus, Phlebotomus-associated flavivirus (PAFV), and co-cell fusing agent virus & PAFV detection, respectively.

Routine mosquito surveillance was conducted in Jeddah from January to June in 2016. The main purpose was to collect mosquitoes from various vulnerable municipalities for speciation and detection of viruses, especially DENV and other arboviruses that might be associated with mosquito species (family Culicidae) present in Jeddah. Mosquitoes were collected with Black Hole strap (BioTrap Co., Ltd, Seoul. S. Korea) which uses UV-LED lamps and photocatalysis to permanently produce carbon dioxide (CO_2_) to lure mosquitoes and a fan to suck them up into the collection bag. The collection was carried out over a 24-h cycle from Sunday to Thursday and transferred in air-conditioned vehicles to the laboratory for sorting at 9:00–11:00 AM daily. The specimens were placed on ice and identified using standard taxonomic keys (Mattingly and Knight, 1956; Knight and Stone, 1977; Harbach and Knight, 1980; Harbach, 1985; Reinert, 2000; Rueda, 2004; Al-Ahmad et al., 2011). Ambiguous specimens were confirmed by molecular methods (Fang et al., 2017). Owing to the large number of adult mosquitoes collected daily, the mosquitoes were randomly sampled by species, date, and collection locality and pooled with a maximum of 20 per pool. Pooled mosquitoes were stored in 2-ml sterile Eppendorf tubes and frozen at −86°C for further nucleic acid extraction. As a preliminary mosquito-borne virus survey, a total of 450 *Ae. aegypti* in 25 pools from 12 municipalities and a total of 407 *Cx. quinquefasciatus* in 24 pools from 10 municipalities in Jeddah were randomly selected among sentinel sites and processed for virus detection. Survey sites are shown in **Figure 1**, from a map generated by using ArcGIS 10.1 ArcMap software (ESRI, Redlands, CA, USA).

### RNA Extraction and Target Gene Sequencing

Pools of mosquitoes were transferred into new tubes containing 450 µl of TRIzol (Invitrogen, Carlsbad, CA, USA) and homogenized in a frozen block using a Mixer Mill (Jingxin, Shanghai, China) with one 3-mm and one 5-mm stainless-steel bead added. The samples were then centrifuged at full speed (12,700 × *rpm*) for 10 mins at 4°C. Then, 350 µl of the supernatant from each homogenate was added to the processing cartridge and placed into the MagNA Pure 96 System (Roche, Basel, Switzerland) for automated RNA extraction as described in a previous study (Fang et al., 2018). First-strand cDNA was synthesized by reverse transcription polymerase chain reaction (PCR) using the PrimeScript RT reagent kit with gDNA eraser (TAKARA BIO, Shiga, Japan). After the RT reactions, PCR amplification targeting mosquito 18S rRNA was implemented with the primers 18S417 and 18S920c (Hoffmann et al., 2004) to verify RNA integrity in each pool. If the control amplification was successful, the cDNA was amplified by semi-nested PCR using a set of primers (cFD2 and MAMD, cFD2 and FS778) for detection of the partial *NS5* gene of flaviviruses as reported previously (Scaramozzino et al., 2001). Alphavirus and orthobunyavirus in mosquito samples were amplified using the primer sets α6533f/α6999c (Bryant et al., 2005) and BCS82C/BCS332V (Kuno et al., 1996), respectively. The amplified products were separated by agarose gel electrophoresis, then purified and sequenced in both directions by Sangon Biotech (Shanghai, China). Sequences were compared with those available in the GenBank database using the BLAST program.

### Whole-Genome Sequencing

For further analysis of the molecular characteristics and possible pathogenetic mechanisms of the viruses, the Primer Premier 5.0 (Premier Biosoft International, Palo Alto, CA, USA) was used to design primers for amplifying the complete genome of DENV and CxFV. DENV whole-genome sequencing was designed using the genomic sequence of an Indian isolate (RGCB921/2011) as reference. To sequence the full-length genome of CxFV, primers were designed from the Chinese strain PA3_17-6E-P-Cxp-C-1-3 (MN318426). The resulting PCR products were sequenced and subsequently used to design new Saudi-isolate-specific primers.

### Comparison of Virus-Deduced Amino Acid Sequences

The whole genomes of DENV and CxFV sequenced in this study were translated into amino acid sequences and aligned with other homologous sequences retrieved from GenBank using MEGA v7.0 [31]. Amino acid substitutions unique to the newly sequenced strains and those different from their closest sequences were observed.

### Phylogenetic Analysis

Multiple sequence alignments were generated using the virus sequences obtained in the present study and homologous sequences were retrieved from GenBank using ClustalW2 (Larkin et al., 2007) with default settings and were manually adjusted as necessary. Neighbor-joining trees were established following the Kimura’s two-parameter distance model (Kimura, 1980) with 1,000 bootstrap replications using MEGA v7.0 (Kumar et al., 2016). Based on the Akaike Information Criterion, the best-fit model for the alignment was determined using Modeltest 3.7 in cooperation with PAUP* v4.0b10 (Wilgenbusch and Swofford, 2003). Consequently, maximum likelihood and Bayesian likelihood trees were constructed under the GTR+I+G model for the DENV-2 whole genome, DENV-2 *E* gene, and CxFV whole genome, and the TrN+I+G model for the ISFV *NS5* gene. The neighbor-joining and maximum likelihood trees were constructed using MEGA v7.0 with 1,000 bootstraps. The Bayesian tree was constructed with MrBayes v3.2.1 (Ronquist et al., 2012) on the CIPRES portal (www.phylo.org/) (Miller et al., 2010) and run for 10 million generations, with the first 25% of generations discarded as burn-in. The trees were visualized using Figtree v1.4.2 (http://tree.bio.ed.ac.uk/software/figtree/).

### Infection Rate Calculation

The size of the pools of collected mosquitoes varied considerably; therefore, infection rates were calculated using bias-corrected maximum likelihood estimation (MLE) and minimum infection rate (MIR) using the Excel add-in PooledInfRate v.4 statistical software package (Biggerstaff, 2006). The rates are expressed as the number of infected mosquitoes per 1,000 collected mosquitoes.

## Results

### Mosquito Adult Species Collected

**Figure 2:**
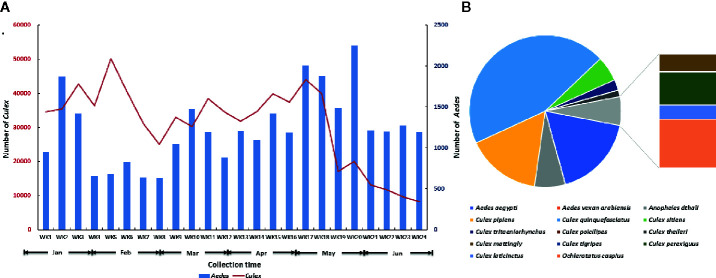
Summary of adult mosquitoes collected at Jeddah in the first half of 2016. **(A)** Seasonal dynamics of *Aedes* and *Culex* mosquitoes. **(B)** Species composition of mosquito in identified mosquitoes.

A total of 29,678 *Aedes* spp. and 739,863 *Culex* spp. were collected at Jeddah from January to June in 2016 (**Figure 2A**). Fourteen species belonging to four genera were found in the 2,499 identified mosquitoes (**Figure 2B**). Among them, *Cx. quinquefasciatus* accounted for 44.78% (1,119/2,499), *Ae. aegypti* accounted for 17.65% (441/2,499), *Cx. pipiens* accounted for 15.85% (396/2,499), *Anopheles dthali* accounted for 6.56% (164/2,499), and *Cx. sitiens* accounted for 5.36% (134/2,499). Nine other species, *Ae. vexan arabiensis*, *Cx. tritaeniorhynchus*, *Cx. poicilipes*, *Cx. theileri*, *Cx. mattingly*, *Cx. tigripes*, *Cx. perexiguus*, *Cx. laticinctus*, and *Ochlerotatus caspius* were collected, but in extremely limited numbers.

### Detection of Flavivirus in Mosquito Pools Collected in Jeddah

Twenty-five pools of *Ae. aegypti* from 12 municipalities and 24 pools of *Cx. quinquefasciatus* from 10 municipalities were randomly selected for virus detection. Control amplifications of the 18S rRNA mosquitoes were successful in all detected pools. The BLAST homology results showed that 14 pools were positive for flavivirus *NS5*, whereas neither alphavirus nor orthobunyavirus were detected. One pool of *Ae. aegypti* was DENV-2 positive, two pools of *Ae. aegypti* were Phlebotomus-associated flavivirus (PAFV) positive and one pool of *Ae. aegypti* was co-positive for PAFV and CFAV. CxFV was detected in 10 pools of *Cx. quinquefasciatus*. All generated sequences were deposited in GenBank (GenBank accession numbers: MN294937–MN294953). Details regarding virus species, host species, collection information, and GenBank accession numbers are listed in **Table 1**. The collection locations of flavivirus-positive pools are shown in **Figure 1**.

**Table 1:** Summary of the flaviviruses detected from different mosquito pools, captured in Jeddah, Saudi Arabia, in the first half of 2016.

Strain	Virus	Host	Collection date	Collection site	Geographic location		GenBank ID	
					Municipality	District	*NS5*	Whole genome
**SA-JD-AS-BM4-16-2-A9**	DENV-2	*Aedes aegypti*	2-Feb-2016	building under-construction	Al-Sharafyia	Bani-Malek-4	MN294939	MN294937
**SA-JD-AJ-AA-16-2-A13**	PAFV	*Ae. aegypti*	25-Feb-2016	building under-construction	Al-Janoub	Al-Ajwad	MN294940	
**SA-JD-JA-AK-16-9-A16**	PAFV	*Ae. aegypti*	9-Mar-2016	building under-construction	Jeddah Aljadeeda	Al-Khalidyia-2	MN294941	
**SA-JD-JA-AM-16-4-A22**	PAFV	*Ae. aegypti*	19-Apr-2016	car tire workshop	Al-Matar	Al-Nuzha-2	MN294942	
**SA-JD-JA-AK-16-9-A16**	CFAV	*Ae. aegypti*	9-Mar-2016	building under-construction	Jeddah Aljadeeda	Al-Khalidyia-2	MN294943	
**SA-JD-Th-Th-16-2-C1**	CxFV	*Culex quinquefasciatus*	12-Jan-2016	human dwelling (house)	Thuwal	Thuwal	MN294944	
**SA-JD-AJ-PFN-16-6-C2**	CxFV	*Cx. quinquefasciatus*	6-Jan-2016	human dwelling (house)	Al-Janoub	Prince Fawaz North	MN294945	
**SA-JD-JA-AS-16-1-C6**	CxFV	*Cx. quinquefasciatus*	18-Jan-2016	building under-construction	Jeddah Aljadeeda	Al-Shatea-1	MN294946	
**SA-JD-AJ-AJ-16-1-C7**	CxFV	*Cx. quinquefasciatus*	31-Jan-2016	building under-construction	Al-Janoub	Al-Jawhara	MN294947	MN294938
**SA-JD-Ta-AS-16-2-C11**	CxFV	*Cx. quinquefasciatus*	29-Feb-2016	dwelling	Tayibah	Al-Salehyia	MN294948	
**SA-JD-Ta-AH-16-3-C12**	CxFV	*Cx. quinquefasciatus*	3-Mar-2016	botanical nursery	Tayibah	Al-Hamdanyia	MN294949	
**SA-JD-Ub-AN-16-3-C13**	CxFV	*Cx. quinquefasciatus*	3-Mar-2016	human dwelling (house)	Ubhor	Al-Naeem-1	MN294950	
**SA-JD-AJ-AJ-16-4-C14**	CxFV	*Cx. quinquefasciatus*	4-Apr-2016	building under-construction	Al-Jameaa	Al-Jameaa	MN294951	
**SA-JD-Ub-AS-16-4-C16**	CxFV	*Cx. quinquefasciatus*	19-Apr-2016	building under-construction	Ubhor	Al-Shatea-6	MN294952	
**SA-JD-JA-AK-16-5-C19**	CxFV	*Cx. quinquefasciatus*	15-May-2016	building under-construction	Jeddah Al-Jadeeda	Al-Khalidyia-2	MN294953	

CFAV, cell fusing agent virus; CxFV, Culex flavivirus; DENV, dengue virus; NS5, non-structural 5; PAFV, Phlebotomus-associated flavivirus.

### Sequence Analysis and Phylogenetic Characterization of the DENV-2 Strain Isolated From Jeddah

**Figure 3:**
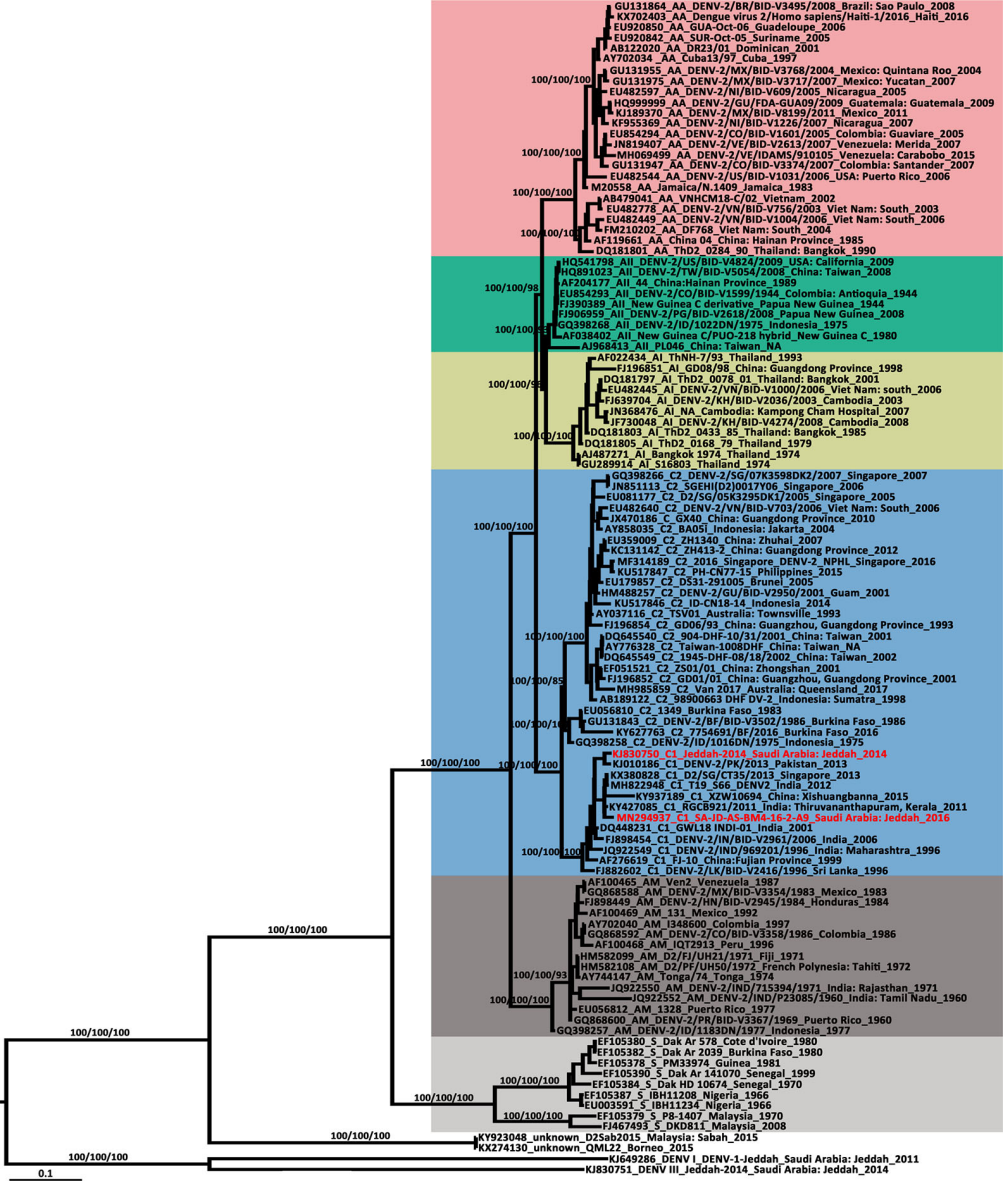
Phylogenetic tree generated by Bayesian analysis of dengue virus 2 (DENV-2) complete genome. The GenBank accession number, virus genotype, strain, collection country and year are noted. The DENV-2 sequences from Saudi Arabia are marked in red. Bootstrap values (1,000 replicates, not shown for less than 75%) of Bayesian analyses, maximum likelihood and neighbor-joining are shown above the main lineages. The scale-bar indicates 0.1 substitutions per site. Sequences referable to the same genotype are shaded with the same colors in the tree. AA, American/Asian; AI, Asian 1; AII, Asian 2; AM, American; C, Cosmopolitan; S, Sylvatic.

**Figure 4:**
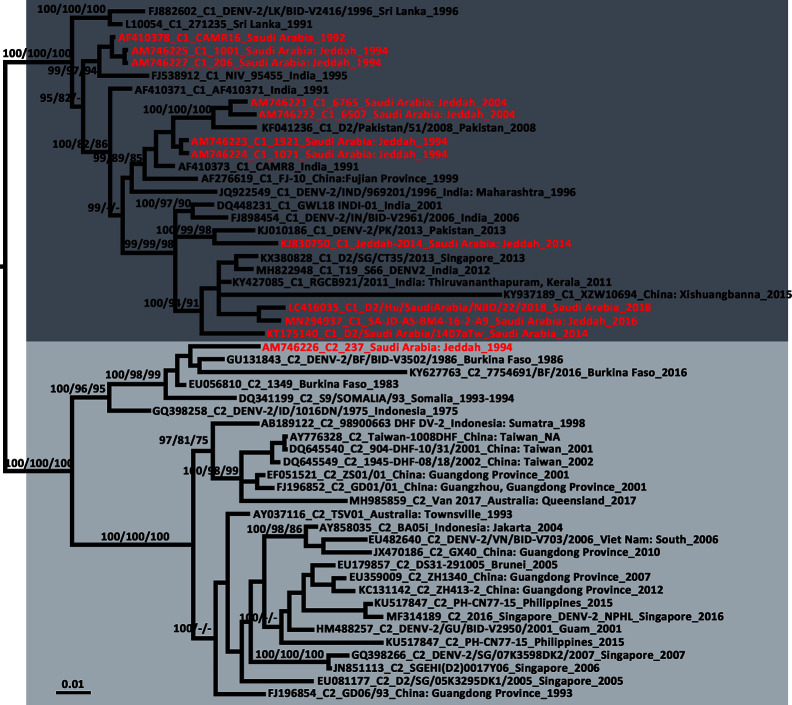
Phylogenetic tree generated by Bayesian analysis of dengue virus 2 (DENV-2) Cosmopolitan genotype complete envelope gene. The GenBank accession number, virus sub-genotype, strain, collection country and year are noted. The DENV-2 sequences from Saudi Arabia are marked in red. Bootstrap values (1,000 replicates, not shown for less than 75%) of Bayesian analyses, maximum likelihood and neighbor-joining are shown above/below the main lineages. The scale-bar indicates 0.01 substitutions per site. Dark gray and light gray indicate DENV-2 Cosmopolitan 1 sub-genotype and DENV-2 Cosmopolitan 2 sub-genotype, respectively. C1, Cosmopolitan 1 sub-genotype; C2, Cosmopolitan 2 sub-genotype.

The newly detected DENV-2 strain was isolated from an *Ae. aegypti* pool collected from the Bani-Malek-4 District, Al-Sharafyia municipality, Jeddah, in February 2016. The length of the whole genome of DENV-2 (SA-AS-BM4-16-2-A9 strain, MN294937) was 10,723 nt, with an open reading frame coding for 3,392 amino acids flanked by 96 nt and 451 nt at the 5′UTR and 3′UTR, respectively. The complete genome was amplified by a total of 17 pairs of overlapping primers (see Supplemental Material **Table S1**). In the BLAST results, the complete genome shared the highest nucleotide identity (98.91%) with the Indian isolate RGCB921/2011 (KY427085) collected in 2011, whereas the genome was slightly distant from that detected in Jeddah in 2014 (Jeddah-2014 strain, KJ830750) with an identity of 97.19%. In the phylogenetic tree based on DENV-2 complete genomes of 106 strains from various sources, six previously described genotypes (Sylvatic, American, American/Asian, Asian 1, Asian 2, and Cosmopolitan) clustered independently with strong bootstrap support (**Figure 3**). Both DENV-2 strains with complete genomes from Saudi Arabia belonged to the Cosmopolitan genotype. Specifically, the two strains were clustered within the Cosmopolitan 1 (C1) sub-genotype which includes strains from India and Sri Lanka in Southeast Asia and Burkina Faso in West Africa. There were 10 other DENV-2 strains with complete envelope (*E*) gene sequences available in GenBank. When combined with the two DENV-2 strains with whole genomes, in the phylogenetic tree of the DENV-2 *E* gene (**Figure 4**), the Saudi strains fell into two lineages. Except the 237 isolate (AM746226) which was isolated in 1994 clustered in the Cosmopolitan 2 (C2) lineage, the other strains were clustered within the C1 lineage. The 237 isolate was closely clustered with sequences from Burkina Faso, and sister to the strain from Indonesia (GQ398258) which was detected in 1975.

Comparing the deduced amino acid sequences of the SA-JD-AS-BM4-16-2-A9 and Saudi-2014 strains, there were 33 non-synonymous changes distributed in capsid (C, 3 positions), pre-membrane (prM, 2 positions), E (3 positions), non-structure 1 (NS1, 2 positions), NS2A (3 positions), NS2B (1 position), NS3 (3 positions), NS4A (1 position), NS4B (3 positions), and NS5 (11 positions) proteins. Most of the changes were observed in the NS5 region of the viral protein with only 1–3 substitutions observed in other proteins. The details of the differences of each position are provided in Supplemental Material **Table S2**. Among them, five amino acid substitutions were found to be unique in the SA-JD-AS-BM4-16-2-A9 strain, (i.e., different from the other DENV-2 strains), including ^103^Gly→Ser, ^265^Arg→Ile, ^284^Ile→Val, ^3119^Cys→Phe, and ^3307^Val→Gly.

It was concluded that the six substitutions on the E protein inducing the Cosmopolitan genotype were separated from other genotypes (Twiddy et al., 2002). Of these substitutions of the 12 Saudi DENV-2 E protein sequences, substitutions on ^71^Glu→Ala, ^149^His→Asn, and ^462^Ile→Val were observed in all strains. ^390^Asn→Ser and ^164^Ile→Val substitutions were detected in all strains except for the 237 isolate (AM746226), whereas the ^129^Ile→Val substitution was only observed in the 237 isolate. This discrepancy was probably due to the differences between the two sub-genotypes (C1 and C2) of the Cosmopolitan genotype.

### Sequence Analysis and Phylogenetic Characterization of CxFV Genotypes

**Figure 5:**
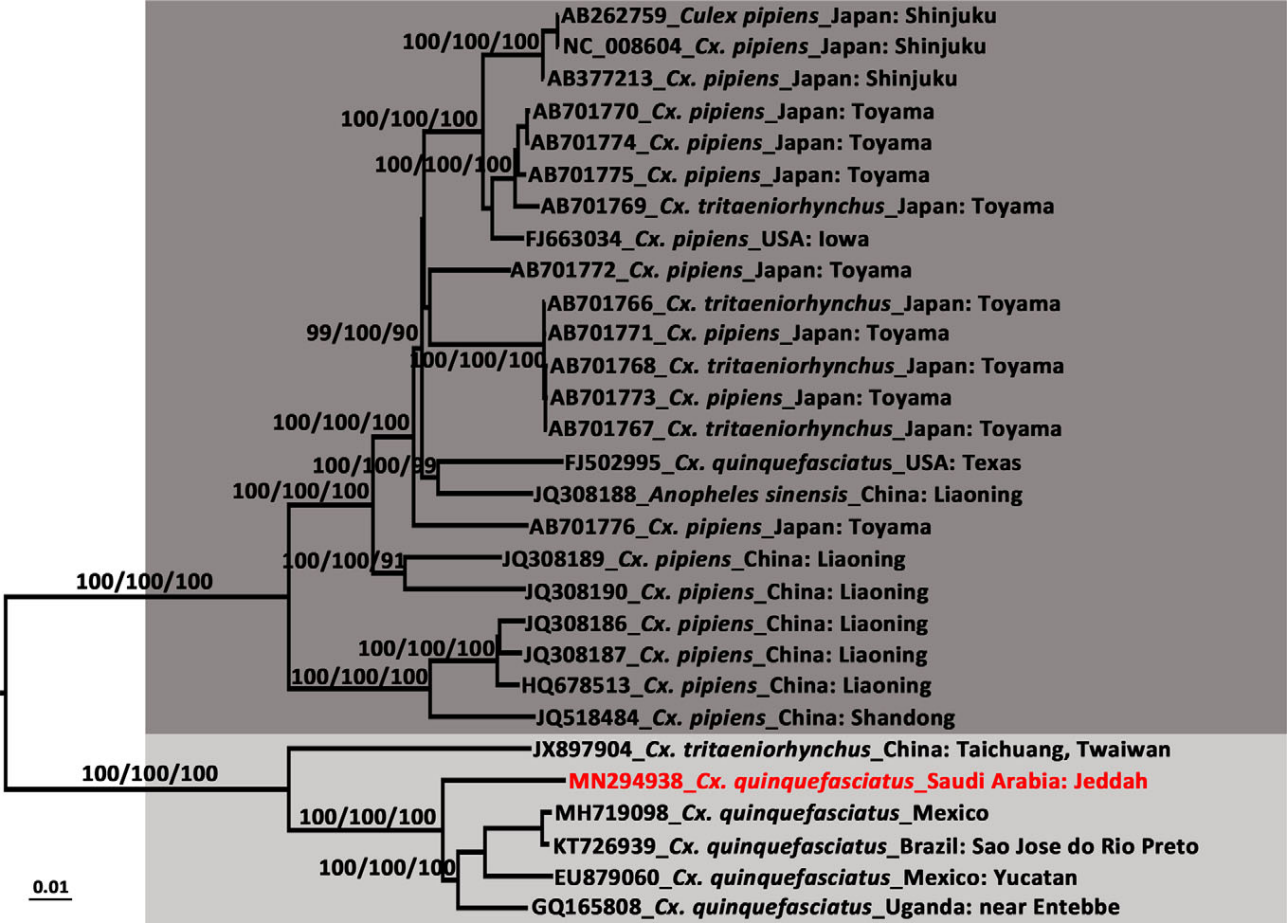
Phylogenetic tree generated by Bayesian analysis of Culex flavivirus (CxFV) complete genome. The GenBank accession number, host species and origin are noted. The CxFV sequences obtained in this study are marked in red. Bootstrap values (1,000 replicates, not shown for less than 75%) of Bayesian analyses, maximum likelihood and neighbor-joining are shown above the main lineages. The scale-bar indicates 0.01 substitutions per site. Dark gray and light gray indicate CxFV Asian/USA genotype and Africa/Caribbean/Latin America genotype, respectively.

**Figure 6:**
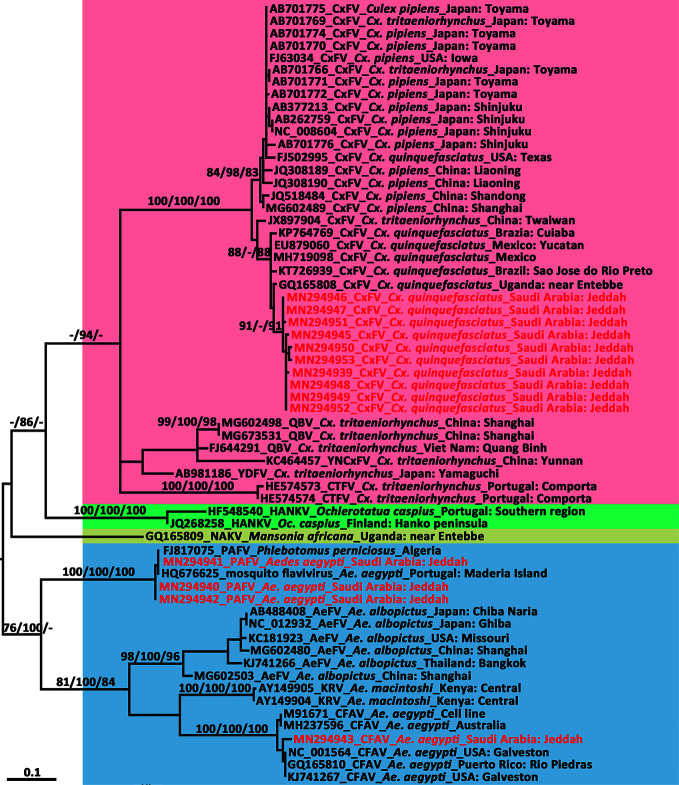
Phylogenetic tree generated by maximum likelihood analysis of insect-specific flavivirus (ISFV) partial non-structural 5 gene. Sequences referable to the same host-genus (except for the Phlebotomus-associated flavivirus) are shown with the same colors in the tree. The ISFV sequences obtained in this study are marked in red. Bootstrap values (1,000 replicates, not shown for less than 75%) of maximum likelihood; Bayesian analyses and neighbor-joining are shown above the main lineages. The bar indicates 0.1 substitutions per site. AEFV, Aedes flavivirus; CFAV, cell fusing agent virus; CTFV, Culex theileri flavivirus; CxFV, Culex flavivirus; HANKV, Hanko virus; KRV, Kamiti River virus; NAKV, Nakiwogo virus; PAFV, Phlebotomus-associated flavivirus; QBV, Quang Binh virus; SCxFV, Spanish Culex flavivirus; YDFV, Yamadai flavivirus; YNCxFV, Yunnan Culex flavivirus.

The length of the CxFV (SA-JD-AJ-AJ-16-1-C7 strain, MN294938) whole genome was 10,799 nt, amplified by a total of 11 overlapping primers (Supplemental Material **Table S3**), with an open reading frame coding for 3,362 amino acids flanked by 71 and 639 nt at the 5′UTR and 3′UTR, respectively. It was closely related to the Mexican CxFV-Mex07 strain (MH719098) with a similarity of 97.57%. In the phylogenetic tree of the CxFV whole genome (**Figure 5**), those isolated from Asia and the USA, and those from Africa, the Caribbean, and Latin America formed two separate clades that represented two genotypes. The SA-JD-AJ-AJ-16-1-C7 strain was clustered within the African/Caribbean/Latin American genotype. As expected, all 10 Saudi CxFVs with *NS5* sequences were clustered in the clade of CxFVs from Africa, the Caribbean, and Latin America in the ISFV *NS5* tree (**Figure 6**).

At the deduced polyprotein level, the Saudi-CxFV strain (SA-JD-AJ-AJ-16-1-C7, MN294938) and its most closely related strain from Mexican (CxFV-Mex07, EU879060) exhibited 17 amino acid substitutions evenly distributed in both structural and non-structural proteins, including C (2 positions), prM (1 position), E (2 positions), NS1 (1 position), NS2A (1 position), NS2B (2 positions), NS3 (3 positions), NS4B (2 positions), and NS5 (3 positions) proteins (Supplemental Material **Table S4**). Among them, eight substitutions were unique in the Saudi CxFV strain: ^3^Lys→Arg, ^325^Asn→Ser, ^868^Ser→Ala, ^1285^Ser→Phe, ^1468^Ala→Val, ^1902^Arg→Thr, ^2404^Ser→Ala, and ^2983^Leu→Phe.

### Phylogenetic Analysis of PAFV and CFAV Sequences

We compared the 261-nt *NS5* sequence of the three flavivirus-positive pools from *Ae. aegypti* against genomic sequences in GenBank using BLAST. The small *NS5*-specific sequence fragments of the three flavivirus pools detected in *Ae. aegypti* showed over 99.36% identity with two short sequences amplified from *Ae. aegypti* (PoMoFlav_R1026 strain, HQ676625, 180 nt) and the sand fly *Phlebotomus perniciosus* (Alg_F19 strain, FJ817075, 157nt). In the phylogenetic tree of the ISFV *NS5* gene (**Figure 6**), they formed a sister clade with other ISFVs isolated from *Aedes* species. The BLAST homology search results showed that, at the nucleotide level, another *NS5* sequence from one of the three PAFV-positive pools in *Ae. aegypti* exhibited 96.17% identity with the CFAV Galveston strain from the USA (NC_001564). In the phylogenetic tree of the ISFV *NS5* gene (**Figure 6**), this sequence was clustered within the CFAV lineage.

### Infection Rates of Flaviviruses in Mosquitoes

The infection rates of flaviviruses in mosquitoes from Jeddah, estimated by bias-corrected MLE and MIR, are shown in **Table 2**. The overall MLE values (with 95% confidence intervals) of DENV, PAFV, and CFAV, expressed as the number of infected mosquitoes per 1,000 *Ae. aegypti*, were 2.22 (0.13–10.80), 6.93 (1.84–18.84), and 2.22 (0.13–10.80), respectively. The MLE of the CxFV per 1,000 in *Cx. quinquefasciatus* was 30.39 (15.75–54.61).

**Table 2:** Maximum likelihood estimation (MLE) and minimum infection rate (MIR) of flavivirus in mosquitoes from Jeddah, Saudi Arabia in the first half 2016.

Detected virus	No. individuals	No. pools	No. PP	PP rate (%)	MLE (95% CI)	MIR (95% CI)
***Aedes aegypti***						
**DENV-2**	450	25	1	4.00	2.22 (0.13–10.80)	2.22 (0.00–6.57)
**PAFV**	450	25	3	12.00	6.93 (1.84–18.84)	6.67 (0.00–14.19)
**CFAV**	450	25	1	4.00	2.22 (0.13–10.80)	2.22(0.00–6.57)
***Culex quinquefasciatus***						
**CxFV**	407	24	10	41.67	30.39 (15.75–54.61)	24.57 (9.53–39.61)

CFAV, cell fusing agent virus; CI, confidence interval; CxFV, Culex flavivirus; DENV, dengue virus; PAFV, Phlebotomus-associated flavivirus; PP, positive pool.

## Discussion

DENV-2 consists of six genotypes: sylvatic, American, Cosmopolitan, Asian 1/Asian 2, and Asian/American, in evolutionary order (Waman et al., 2016). In the phylogenetic tree of the DENV-2 whole genome (**Figure 3**), the SA-JD-AS-BM4-16-2-A9 strain belonged to the DENV-2 Cosmopolitan genotype and was further classified into the C1 sub-genotype. The C1 sub-genotype mainly circulates in the Indian subcontinent (Kumar et al., 2010). Based on the tree, this sub-genotype probably originated from India, spreading eastward to the Yunnan and Fujian Provinces, China, southward to Sri Lanka and Singapore, and radiating westward toward Pakistan and Saudi Arabia. The other sub-genotype of the Cosmopolitan genotype is the C2 sub-genotype, which has been reported in a diverse range of geographical areas worldwide (Twiddy et al., 2002).

From the tree based on the *E* gene of DENV-2 Cosmopolitan genotype (**Figure 4**), 12 strains that were detected in Saudi Arabia using the complete *E* gene available in GenBank were dispersed in five different clusters of the Cosmopolitan genotype. The 12 strains were collected over 25 years. Eleven of them belonged to the C1 sub-genotype, whereas one was classified to the C2 sub-genotype. The tree indicates at least five independent DENV-2 introduction events into Saudi Arabia (**Figure 4**). Three of them occurred before 1994 and two occurred before 2014. Almost all of them were related to the strains from the Indian subcontinent, supporting the speculation that the prevalence of DENV in Saudi Arabia is mainly associated with religious pilgrimages and Umrah (Alhaeli et al., 2016; El-Kafrawy et al., 2016; Al-Saeed et al., 2017). Jeddah governorate is a major coastal city and harbor on the Red Sea. The newly-detected strain SA-JD-AS-BM4-16-2-A9 was obtained from mosquitoes collected in a building under construction in the Bani-Malek-4 District, Al-Sharafyia Municipality, which is only 15 km away from the King Abdulaziz International Airport. Notably, in the phylogenetic tree, it is clustered with the strain D2/Hu/SaudiArabia/NIID/22/2018 (LC416035) detected from a Japanese traveler returning from Saudi Arabia in 2018 (Matsui et al., 2019), and was sister to the strain D2/Saudi Arabia/1407aTw_Saudi Arabia (KT175140) isolated from a suspected dengue patient from Taiwan, China, in 2014, who had traveled to Saudi Arabia (Chang et al., 2016). It can be inferred that this ‘newly’ detected DENV-2 strain was probably introduced by pilgrims or working expats in Saudi Arabia before 2014, subsequently evolving and circulating in local areas. This strain and its relatives not only infected local vectors but also spread from Saudi Arabia to other countries before it was even detected in Saudi Arabia, reflecting the lack of mosquito-borne disease surveillance in Saudi Arabia. It is important to note that seasonal pilgrimage and Umrah are important factors for introduction of DENV into Saudi Arabia. However, millions of expats from Africa and Asian dengue-endemic countries (Southeast Asia and Indian subcontinent) live and work in Saudi Arabia, which is also a major mechanism of virus introduction/exportation.

The potentially high transmission capacity of the strain SA-JD-AS-BM4-16-2-A9 is indicated by the discovery of the earliest detected strain in this cluster in India in 2011. In the next four years, its relatives spread to Singapore (2013), Saudi Arabia (2014), and China (2015) (**Figure 4**). Among the four serotypes, DENV-2 has been associated with severe dengue cases and is usually the most frequent cause of dengue outbreaks worldwide (Ricohesse, 2003; Cologna et al., 2005). However, only *E* gene sequences for both strains exported from Saudi Arabia were available in GenBank. No unique substitutions on the E protein of these three strains were observed. Full viral genome sequencing is an important tool for understanding both viral evolution and the mechanisms underlying viral virulence (Christenbury et al., 2010). Thus, further research is required to clarify whether the unique substitutions of the strain SA-JD-AS-BM4-16-2-A9 detected here at the whole genome level are functionally important in terms of increasing virulence, affecting dispersal patterns, or increasing the selection pressure on the host, vector, or both. The substitution of ^390^Asn→Ser on the E protein was observed in all 11 DENV-2 C1 strains from Saudi Arabia. This substitution has previously been implicated in altering virulence and cellular tropism, and may lead to changes in transmissibility (Sánchez and Ruiz, 1996; Leitmeyer et al., 1999; Twiddy et al., 2002).

In the phylogenetic tree of the DENV-2 whole genome (**Figure 3**), each genotype formed a distinct clade, except two strains from Borneo (D2Sab2015 strain, KY923048; and QML22 strain, KX274130). Both were imported to Australia in 2015 and were temporarily classified as DENV-2 based on serologic tests but are highly divergent and basal to all other genotypes of DENV-2 (Liu et al., 2016; Pyke et al., 2017). Their phylogenetic position, as confirmed in the present study, is distant from human and sylvatic strains. However, both of their nucleotide sequences had ~76.50% identity compared to other DENV-2 strains, which is a bit more than the threshold of sequence differences between serotypes (65–70%) (Green and Rothman, 2006). In addition, in 2014, the most divergent DENV-1 was recorded from a viremic patient who had visited the rainforest of Brunei, Borneo and then returned to Australia (Pyke et al., 2016). Given that a series of highly divergent DENV strains have been found in Borneo in recent years, Borneo probably has a high DENV diversity.

The estimated infection rate of DENV in *Ae. aegypti* was 2.22 per 1,000 individuals in Jeddah. However, this result is probably underestimated because we did not use the homogenate of mosquito pools to infect mosquito cell lines. A relatively low viral load in mosquitoes is difficult to detect by direct RNA isolation. In addition, although the mosquito samples were transported at low temperature, the viral RNA might have degraded with the cycles of freezing and thawing and long-distance transportation.

Insect-specific flaviviruses (ISFVs) are flaviviruses that have been detected in mosquitoes with no-known vertebrate host (Cook et al., 2012). Many ISFVs have been detected in this century (Crabtree et al., 2003; Cook et al., 2006; Hoshino et al., 2007; Crabtree et al., 2009; Chen et al., 2013). There were 14 strains of ISFVs belonging to CxFV, PAFV, and CFAV reported in the present study. Culex flavivirus is the most diverse and prevalent ISFV detected so far and can be divided into two genotypes. One is prevalent in Asia and the USA and is mainly detected in *Cx. pipiens* but is also found in *Cx. quinquefasciatus*, *Cx. tritaeniorhynchus*, and *An. sinensis*; the other is distributed in Africa, the Caribbean, and Latin America, sharing the same host (*Cx. quinquefasciatus*) (Bittar et al., 2016). Sequence alignment and phylogenetic analysis of the *NS5* gene of 10 Saudi CxFV strains showed that they have more than 96.56% (96.56–99.62%) nucleotide identity and were clustered in the clade that included strains from Africa, the Caribbean, and Latin America. Here, the African/Caribbean/Latin American genotype was detected in the Middle East, indicating that this CxFV genotype could be transmitted and sustained in geographically distant countries. The prevalence of CxFV in *Cx. quinquefasciatus* was high in Jeddah. Among the 24 pools of *Cx. quinquefasciatus*, 10 were positive for CxFV. The MLE infection rate was 30.39 per 1,000 mosquitoes, which is much higher than that in the *Cx. pipiens* (1.34) population from Shanghai, China (Fang et al., 2018). The MIR (24.57) of CxFV in Jeddah is comparable to that in the Yucatan Peninsula of Mexico (20.8) (Farfan-Ale et al., 2009), whereas it was 4.7 per 1,000 in Guatemala, which is near the Yucatan Peninsula (Morales-Betoulle et al., 2008). It has been reported that infection with CxFV may increase the WNV infection rate (Kent et al., 2010; Newman et al., 2011). Whereas; some other studies showed that prior infection of ISFVs will suppress subsequent replication of mosquito-borne flaviviruses associated with human diseases (Hobsonpeters et al., 2013; Goenaga et al., 2015). Further study is needed to investigate whether the presence of ISFVs represent a potential threat to human or animal health.

Phlebotomus-associated flavivirus was first recorded in samples of *P. perniciosus* collected in Algeria between August 2006 and July 2007 (Moureau et al., 2010). PAFV was named after the genus of its host species, similar to other ISFVs, but is the sole ISFV that was not named after a mosquito genus. Two strains of ‘mosquito flavivirus’ were detected in *Ae. aegypti* from Madeira Island, Portugal in 2010 and 2013 (Osório et al., 2014). Together with the three strains detected in the present study, these strains were clustered as a monophyletic clade distinct from other Aedes flaviviruses based on the phylogenetic analysis of a partial *NS5* gene (**Figure 6**). Since this ISFV was first detected in sand flies, and the name ‘mosquito flavivirus’ would be confused with other flaviviruses, we designated the three new ISFV strains as PAFV. Most ISFV vectors are genus- or species-specific. CxFVs have diverse hosts and are not only restricted to *Culex* species but were also once found in *An. sinensis* (Liang et al., 2015), representing intergeneric infection. Moreover, PAFVs have been detected in both *P. perniciosus* and *Ae. aegypti*, indicating trans-family diffusion. Most of the physiological and ecological dynamics of ISFVs, including their maintenance cycles in nature, remain unknown (Hoshino et al., 2009). PAFVs have been found in the Mediterranean and Red Sea regions (Moureau et al., 2010; Osório et al., 2014), which might indicate that these habitats are suitable for the transmission of PAFV and provides evidence to suggest that the transmission of ISFVs in sympatric species is potentially linked to a common source of infection, especially through feeding (Cook et al., 2009; Calzolari et al., 2016). However, the sequences of PAFVs available in GenBank for strains from Algeria and Portugal are short (<200 nt in length). We did not successfully sequence the whole genome of PAFV, mainly owing to the low concentration of PAFV RNAs in vectors collected from Jeddah and the lack of a reference genome. Nevertheless, it is important to obtain the whole genome of PAFV to further analyze its trans-family diffusion in insects and the underlying infectious mechanisms.

Cell-fusing agent virus was the first ISFV detected, and was found in an *Ae. aegypti* cell line in 1975 (Stollar and Thomas, 1975). A strain of CFAV co-detected with PAFV in a pool of *Ae. aegypti* was observed in the present study. Since the pool contained 18 mosquitoes, it is possible that the two different ISFVs infected two or more individuals of *Ae. aegypti*; however, the possibility that the two ISFVs co-infected the same mosquito cannot be ruled out. It was reported that mosquitoes infected with ISFVs might be more susceptible to pathogenic flaviviruses (Crabtree et al., 2009; Vazquez et al., 2012). Whether the effect of superinfection exists in ISFVs is unclear. Other CFAVs have been found in Galveston, USA and Puerto Rico, and in the present study were detected in Jeddah. Although these CFAV isolates are geographically distant, the latitude of their collection sites is comparable. This indicates that the distribution of CFAVs might be influenced by climate, similar to the two CxFV genotypes (Bittar et al., 2016).

One major limitation of our study is the small sample size of mosquitoes used for virus detection. Among the 49 mosquito pools, 14 were flavivirus-positive, whereas the actual mosquito-borne virus in Jeddah is probably more diverse and of higher prevalence. Since the potential in the supernatant of mosquito homogenate viruses have not been cultivated in mosquito or human cell lines and might degrade during long-term storage for direct RNA extraction, the low concentration or titer of a given virus in mosquito homogenates would influence the results of pathogen detection. In addition, we did not detect any DENV-1, DENV-3, or DENV-4 in the tested samples, possibly owing to the low circulation of these viruses and a small sample size of host mosquitoes, but they might co-circulate in local areas. Wider sampling and virus isolation for mosquito-borne disease surveillance in Jeddah and other regions of Saudi Arabia should be carried out for a more comprehensive understanding of the virus diversity, infection rates, transmission routes, virulence, and pathogen mechanisms in this region.

In conclusion, one human-pathogen DENV and three different ISFVs, including CFAV, PAFV, and CxFV, were detected in local dominant mosquito vector species *Ae. aegypti* and *Cx. quinquefasciatus* of Jeddah, western Saudi Arabia. To the best of our knowledge, this study is the first to report viral detection in field-caught mosquitoes in Saudi Arabia. The newly detected DENV strain belonged to the C1 sub-genotype of DENV-2, possessing five unique amino acid substitutions. The phylogenetic analyses revealed that most introduction events of DENV-2 to Saudi Arabia were related to strains from the Indian subcontinent, indicating that the DENV prevalent in Saudi Arabia is associated with religious pilgrimages and resident working expats from Asian dengue-endemic countries. The phylogenetic analyses further indicated that relatives of the detected DENV-2 strain in Jeddah were probably exported to Taiwan, China and Japan in 2014 and 2018, which suggests that DENV prevalence in Jeddah is probably more complex than previously thought, and will become even worse with the increasing frequency of international travel by air and sea, especially without efficient surveillance of mosquito-borne diseases. Strategies for mosquito-borne pathogen control should not only focus on the pilgrims and travelers from DENV-endemic areas for the potential risk of DENV introduction into Saudi Arabia but also on prevention of the exportation of locally circulating virus genotypes to other countries when these visitors return home. The high prevalence of CxFV in *Cx. quinquefasciatus* from Jeddah suggests that CxFV is widespread in this region, which may increase the risk of infection with WNV (Kent et al., 2010; Newman et al., 2011), representing a potential threat to human or animal health. The preliminary findings reported herein with randomly selected small mosquito samples highlight that wide-ranging, systematic, and continuous molecular monitoring of mosquito-borne circulating viruses in vectors is urgently needed. This would provide a comprehensive understanding of virus diversity, geographic distribution, evolution, shifts in circulating genotypes, and infection rates in Jeddah and other regions of Saudi Arabia and allow for accurate and timely estimations of the true disease burden and prevalence of dengue and other emerging/re-emerging mosquito-borne pathogens. This is essential to support the decision-making process regarding appropriate prevention and control strategies in Saudi Arabia, the Arabian Peninsula, and the whole Middle East region.

## Data Availability Statement

The datasets presented in this study can be found in online repositories. The names of the repository/repositories and accession number(s) can be found in the article/**Supplementary Material**.

## Author Contributions

YF: conceptualization, methodology, writing original draft preparation, and writing review and edited. ET: conceptualization, methodology, investigation, project administration, and writing review and edited. J-BX: methodology and software. YZ: conceptualization, funding acquisition, and writing review and edited. X-NZ: funding acquisition, and wrote—review and edited. EK: conceptualization, methodology, investigation, and writing review and edited. All authors contributed to the article and approved the submitted version.

## Funding

The Special Foundation of Basic Science and Technology Resources Survey of Ministry of Science and Technology of China (grant no. 2017FY101200), the Project of Basic Platform of National Science and Technology Resources of the Ministry of Sciences and Technology of China (No. TDRC-2019-194-30).

## Conflict of Interest

The authors declare that the research was conducted in the absence of any commercial or financial relationships that could be construed as a potential conflict of interest.

The reviewer KK declared a past co-authorship with one of the authors X-NZ to the handling editor.
