# Supplementary material for: Detection of DENV-2 and Insect-Specific Flaviviruses in Mosquitoes Collected From Jeddah, Saudi Arabia
Source: Front Cell Infect Microbiol. 2021 Feb 25;11:626368. doi: 10.3389/fcimb.2021.626368 (PMC7947193; doi:10.3389/fcimb.2021.626368)
Supplement: Supplementary file 1 [file Table_1.docx]

Supplementary Material

# Supplementary Tables

**Table S1. Primer sets used for sequencing the DENV-2 SA-JD-AS-BM4-16-2-A9 strain**

| Primer | Position | Sequence, 5’-3^’a^ | Polarity |
| --- | --- | --- | --- |
| 1-F | 1-24 | AGTTGTTAGTCTACGTGCACCGAC | Sense |
| 1-R | 560-583 | TTGGTGTCTTCACACAGTTCACCAA | Reverse |
| 2-F | 394-414 | AGAACTGCAGGCGTGATCATC | Sense |
| 2-R | 1170-1192 | GTTCTTCATTTAGGCTGGGTTCT | Reverse |
| 3-F | 1006-1028 | GTCTTAGAACATGGAAGCTGCGT | Sense |
| 3-R | 1682-1705 | CTTGGGATCCTAAAACAACAACAT | Reverse |
| 4-F | 1539-1559 | GGAAAATAAGGCTTGGCTGGT | Sense |
| 4-R | 2316-2340 | CATTCCTATCCATGTGATGACAACT | Reverse |
| 5-F | 2180-2200 | TAGGTGACACAGCCTGGGATT | Sense |
| 5-R | 3113-3134 | TCTAGCACTCCATTGCTCCAGA | Reverse |
| 6-F | 2933-2954 | TGAAAGAAAGGCAGGATGTGTT | Sense |
| 6-R | 3565-3590 | ACTGCAACTAGTAATACTGCATGCTT | Reverse |
| 7-F | 3420-3443 | GGAAATCAGACCATTGAAAGAGAA | Sense |
| 7-R | 4031-4050 | CCAATCCGCTTTCTGCTGTG | Reverse |
| 8-F | 3873-3899 | GATTCTCAAGATAGTGAGGAATATGGA | Sense |
| 8-R | 4819-4841 | CTTGGATTCTTTCCAGGCTCTAA | Reverse |
| 9-F | 4653-4674 | AACATTCCACACAATGTGGCAC | Sense |
| 9-R | 5226-5252 | GTTTGGTATCTTATTGGAAGTCCTCTA | Reverse |
| 10-F | 5049-5074 | AGAGATTGAAGATGATATCTTTCGGA | Sense |
| 10-R | 5683-5711 | GTCTTGATATATTCAGAATCAAAGGTCTT | Reverse |
| 11-F | 5558-5581 | CTGGACATGAGTGGGTTACAGATT | Sense |
| 11-R | 6522-6543 | AAGCAATGTCTCCAGGGTCTCT | Reverse |
| 12-F | 6340-6364 | CTCAAAGAATTCAAAGAGTTTGCAG | Sense |
| 12-R | 7284-7309 | GATCATAGGGTATTGGATCTAGGTCA | Reverse |
| 13-F | 7139-7160 | TAACCCTTACAGCAGCCCTTCT | Sense |
| 13-R | 8331-8350 | CGCTTCCGAGGTCAACATCT | Reverse |
| 14-F | 8149-8168 | ATGGAAACGCTGCAAAGGAA | Sense |
| 14-R | 9185-9212 | TTTAAGTCCTCTAGTGTGATTCTTGTGT | Reverse |
| 15-F | 9053-9077 | ATCACTGGTTTTCCAGAGAGAACTC | Sense |
| 15-R | 9467-9488 | TCTTCTGTGGCTGTCAGATGCT | Reverse |
| 16-F | 9274-9298 | TTCAAGTTAACGTACCAAAACAAGG | Sense |
| 16-R | 10402-10421 | AGGCTGCATAGTTTGCTCCA | Reverse |
| 17-F | 10236-10257 | CAGAAGAGAAGAGGAGGAGGCA | Sense |
| 17-R | 10701-10723 | AGAACCTGTTGATTCAACAGCAC | Reverse |

^a^The primers were designed from the India isolate RGCB921/2011 (KY427085).

**Table S2. Comparison on amino acid substitutions between DENV-2 SA-JD-AS-BM4-16-2-A9 strain and DENV-2 Jeddah-2014 strain**

| S.N. | Protein | Mutation sites | Strain | |
| --- | --- | --- | --- | --- |
|  |  |  | SA-JD-AS-BM4-16-2-A9 (MN294937) | Jeddah-2014 (KJ830750) |
| 1 | C | 71 | T | I |
| 2 | C | 92 | L | M |
| 3 | C | 103 | S | G |
| 4 | prM | 130 | R | K |
| 5 | prM | 265 | I | R |
| 6 | E | 284 | V | I |
| 7 | E | 506 | T | I |
| 8 | E | 640 | E | G |
| 9 | NS1 | 862 | T | S |
| 10 | NS1 | 953 | F | S |
| 11 | NS1 | 1099 | K | R |
| 12 | NS2A | 1243 | I | T |
| 13 | NS2A | 1246 | T | S |
| 14 | NS2A | 1301 | V | A |
| 15 | NS2B | 1400 | R | K |
| 16 | NS3 | 1489 | E | G |
| 17 | NS3 | 2010 | E | D |
| 18 | NS3 | 2063 | K | R |
| 19 | NS4A | 2097 | M | L |
| 20 | NS4B | 2262 | V | A |
| 21 | NS4B | 2263 | T | I |
| 22 | NS4B | 2393T | I | V |
| 23 | NS5 | 2569 | M | V |
| 24 | NS5 | 2891 | T | A |
| 25 | NS5 | 2903 | V | I |
| 26 | NS5 | 3122 | S | G |
| 27 | NS5 | 3128 | T | A |
| 28 | NS5 | 3139 | V | A |
| 29 | NS5 | 3167 | N | S |
| 30 | NS5 | 3119 | F | C |
| 31 | NS5 | 3291 | R | G |
| 32 | NS5 | 3307 | G | V |
| 33 | NS5 | 3352 | K | R |

C: capsid; E: envelope; NS: non-structural; prM: pre-membrane.

**Table S3. Primer sets used for sequencing the CxFV SA-JD-AJ-AJ-16-1-C7 strain**

| Primer | Position | Sequence, 5’-3^’a^ | Polarity |
| --- | --- | --- | --- |
| 1-F | 1-22 | TGGTTACACCGCAGATTGGTTA | Sense |
| 1-R | 1181-1200 | CACGATTGTAGGGCTGGGTT | Reverse |
| 2-F | 1050-1069 | ATATATGGCGGCCAATGGAT | Sense |
| 2-R | 2249-2269 | CTTTTCCAGACGAACCCTCCT | Reverse |
| 3-F | 2089-2109 | GCCTGTTGTCTAGCTCGACGA | Sense |
| 3-R | 3314-3337 | ATTGATGTCATCTCGTCCATGTCT | Reverse |
| 4-F | 3174-3194 | GCCAAGAAGTGGTGTTGCAAG | Sense |
| 4-R | 4393-4413 | CCGATACTCCAGCCACGTAGA | Reverse |
| 5-F | 4236-4258 | GATGGAGTGGAATTCACGAACTT | Sense |
| 5-R | 5481-5504 | TGTGGGAACGAATATTATCGTCTT | Reverse |
| 6-F | 5351-5371 | CGTGGTGTACATGAGTGCGAC | Sense |
| 6-R | 6595-6613 | AATCCCAACACCGCAACAA | Reverse |
| 7-F | 6415-6436 | TTGTAGTGGTTTGTCGGATCGT | Sense |
| 7-R | 7712-7733 | CACCAGACGTTGAGTCCATCCT | Reverse |
| 8-F | 7594-7615 | TGAACGAGACAGACAAGGGTGA | Sense |
| 8-R | 8874-8897 | CCACATGTACCAGATAATCCTTGA | Reverse |
| 9-F | 8663-8684 | CATGAACAACGTAAAAAGCGGA | Sense |
| 9-R | 9781-9799 | GCCGACGTGATAGCAGCAA | Reverse |
| 10-F | 9580-9602 | GTTCCCATCACTACCATCCTCTC | Sense |
| 10-R | 10131-10151 | GTCGTCAGTTGGAGCTCGGTA | Reverse |
| 11-F | 9982-10007 | AGGACATAAACTGTGGAAGTTTGATC | Sense |
| 11-R | 10780-10799 | GCCCGCAACAAGTCTCCTAA | Reverse |

^a^The primers were designed from the China strain PA3_17-6E-P-Cxp-C-1-3 (MN318426).

**Table S4. Amino acid substitutions observed in CxFV strain SA-JD-AJ-AJ-16-1-C7 compared to the most closely related strain**

| S.N. | Protein | Mutation sites | strain | |
| --- | --- | --- | --- | --- |
|  |  |  | SA-JD-AJ-AJ-16-1-C7 (MN294938) | CxFV-Mex07 (EU879060) |
| 1 | C | 3 | R | K |
| 2 | C | 127 | S | T |
| 3 | prM | 223 | V | A |
| 4 | E | 325 | S | N |
| 5 | E | 668 | G | K |
| 6 | NS1 | 868 | A | S |
| 7 | NS2A | 1285 | F | S |
| 8 | NS2B | 1328 | L | F |
| 9 | NS2B | 1377 | I | T |
| 10 | NS3 | 1468 | V | A |
| 11 | NS3 | 1782 | D | A |
| 12 | NS3 | 1902 | T | R |
| 13 | NS4B | 2320 | V | I |
| 14 | NS4B | 2404 | A | S |
| 15 | NS5 | 2983 | F | L |
| 16 | NS5 | 3022 | N | T |
| 17 | NS5 | 3290 | L | Q |

C: capsid; E: envelope; NS: non-structural; prM: pre-membrane.
